# Deep learning representations to support COVID-19 diagnosis on CT slices

**DOI:** 10.7705/biomedica.5927

**Published:** 2022-03-01

**Authors:** Josué Ruano, John Arcila, David Romo-Bucheli, Carlos Vargas, Jefferson Rodríguez, Óscar Mendoza, Miguel Plazas, Lola Bautista, Jorge Villamizar, Gabriel Pedraza, Alejandra Moreno, Diana Valenzuela, Lina Vásquez, Carolina Valenzuela-Santos, Paúl Camacho, Daniel Mantilla, Fabio Martínez

**Affiliations:** 1 BIVL2ab Biomedical Imaging, Vision and Learning Laboratory, Escuela de Ingeniería de Sistemas e Informática, Universidad Industrial de Santander, Bucaramanga, Colombia Universidad Industrial de Santander BIVL2ab Biomedical Imaging, Vision and Learning Laboratory Escuela de Ingeniería de Sistemas e Informática Universidad Industrial de Santander Bucaramanga Colombia; 2 Clínica FOSCAL, Fundación Oftalmológica de Santander, Bucaramanga, Colombia Clínica FOSCAL Fundación Oftalmológica de Santander Bucaramanga Colombia; 3 Facultad de Ingeniería, Universidad de Los Andes, Mérida, Venezuela Universidad de Los Andes Facultad de Ingeniería Universidad de Los Andes Mérida Venezuela

**Keywords:** Coronavirus infections/diagnosis, tomography, X-ray computed, deep learning, infecciones por coronavirus/diagnóstico, tomografía computarizada por rayos X, aprendizaje profundo

## Abstract

**Introduction:**

The coronavirus disease 2019 (COVID-19) has become a significant public health problem worldwide. In this context, CT-scan automatic analysis has emerged as a COVID-19 complementary diagnosis tool allowing for radiological finding characterization, patient categorization, and disease follow-up. However, this analysis depends on the radiologist’s expertise, which may result in subjective evaluations.

**Objective:**

To explore deep learning representations, trained from thoracic CT-slices, to automatically distinguish COVID-19 disease from control samples.

**Materials and methods:**

Two datasets were used: SARS-CoV-2 CT Scan (Set-1) and FOSCAL clinic’s dataset (Set-2). The deep representations took advantage of supervised learning models previously trained on the natural image domain, which were adjusted following a transfer learning scheme. The deep classification was carried out: (a) via an end-to-end deep learning approach and (b) via random forest and support vector machine classifiers by feeding the deep representation embedding vectors into these classifiers.

**Results:**

The end-to-end classification achieved an average accuracy of 92.33% (89.70% precision) for Set-1 and 96.99% (96.62% precision) for Set-2. The deep feature embedding with a support vector machine achieved an average accuracy of 91.40% (95.77% precision) and 96.00% (94.74% precision) for Set-1 and Set-2, respectively.

**Conclusion:**

Deep representations have achieved outstanding performance in the identification of COVID-19 cases on CT scans demonstrating good characterization of the COVID-19 radiological patterns. These representations could potentially support the COVID-19 diagnosis in clinical settings.

## Abstract (es)

**Introducción.:**

La enfermedad por coronavirus (COVID-19) es actualmente el principal problema de salud pública en el mundo. En este contexto, el análisis automático de tomografías computarizadas (TC) surge como una herramienta diagnóstica complementaria que permite caracterizar hallazgos radiológicos, y categorizar y hacer el seguimiento de pacientes con COVID-19. Sin embargo, este análisis depende de la experiencia de los radiólogos, por lo que las valoraciones pueden ser subjetivas.

**Objetivo.:**

Explorar representaciones de aprendizaje profundo entrenadas con cortes de TC torácica para diferenciar automáticamente entre los casos de COVID-19 y personas no infectadas.

**Materiales y métodos.:**

Se usaron dos conjuntos de datos de TC: de SARS-CoV-2 CT (conjunto 1) y de la clínica FOSCAL (conjunto 2). Los modelos de aprendizaje supervisados y previamente entrenados en imágenes naturales, se ajustaron usando aprendizaje por transferencia. La clasificación se llevó a cabo mediante aprendizaje de extremo a extremo y clasificadores tales como los árboles de decisiones y las máquinas de soporte vectorial, alimentados por la representación profunda previamente aprendida.

**Resultados.:**

El enfoque de extremo a extremo alcanzó una exactitud promedio de 92,33 % (89,70 % de precisión) para el conjunto 1 y de 96,99 % (96,62 % de precisión) para el conjunto-2. La máquina de soporte vectorial alcanzó una exactitud promedio de 91,40 % (precisión del 95,77 %) para el conjunto-1 y del 96,00 % (precisión del 94,74 %) para el conjunto 2.

**Conclusión.:**

Las representaciones profundas lograron resultados sobresalientes al caracterizar patrones radiológicos usados en la detección de casos de COVID-19 a partir de estudios de TC y demostraron ser una potencial herramienta de apoyo del diagnóstico.

Coronavirus disease 2019 (COVID-19) emerges nowadays as the major public health problem worldwide and it is the third coronavirus outbreak in the last two decades ^1^^,^^2^. According to the Center for Systems Science and Engineering (CSSE), until June 2021, there were 176’349,164 confirmed cases worldwide ^3^ and in Colombia, there were 3’777,600 confirmed cases and 96.366 deaths associated with the disease ^3^. COVID-19 is a disease caused by the severe acute respiratory syndrome coronavirus 2 (SARS- CoV-2) ^2^^,^^4^, which belongs to the betacoronavirus genus; it takes a mean of 5 days for incubation and its initial manifestations are similar to those by another respiratory tract virus ^2^^,^^4^. The infection may progress to the lower respiratory tract with symptoms ranging from dyspnea with progressive oxygen desaturation to severe pneumonia, usually present in the second or third week. In advanced stages, there is the risk of acute respiratory distress syndrome ^2^^,^^4^ requiring specialized clinical interventions in the intensive care units to avoid possible asepsis, septic shock, and even death. Such requirements can overwhelm public health systems limiting the adequate provision of services and causing increased mortality in the affected population ^5^.

The early detection of the infection is the most effective strategy to treat and follow patients, as well as to decrease disease transmission allowing quick reactions such as timely lockdowns ^6^. The gold standard test for COVID-19 diagnosis is the reverse transcription-polymerase chain reaction (RT-PCR) ^7^, however, a high false-negative rate has been reported ranging between 20% and 67% ^8^. This variability may respond to the difficulty of obtaining high-quality samples and the timing of testing ^4^. A recent study estimating the sensitivity of the RT-PCR on 1,194 inpatients and 1,814 outpatients concluded that it was moderate at best ^9^. The authors reported that when taking into account highly suspicious cases (which never tested positive), the estimated sensitivity (95% CI) was 67.5% (62.9-71.9%) for inpatients, 34.9% (31.4-38.5%) for outpatients, and 47.3% (44.4-50.3%) for all. Additionally, the delay in the result of the RT-PCR test interferes with an early diagnosis of the disease ^10^. For those reasons, radiological- image analyses have emerged as a powerful technique to support the diagnosis and characterize symptomatic cases and a complementary tool in the personalized characterization of the disease ^11^^-^^14^. Among others, the analysis of radiological visual patterns over CT scans allows to stratify the disease, define specific treatments, and follow the evolution from a personalized perspective. In a study by Bai, *et al.*^15^, a group of radiologists with different levels of experience were evaluated at the task of differentiating COVID-19 disease from viral pneumonia on thoracic CT-scans obtaining a sensibility ranging between 56% and 98%. Nevertheless, the same study showed a low specificity (25%) among radiologists with little experience ^15^^,^^16^. Knowing that the COVID-19 visual patterns on CT scans are very similar to other lung infections, experts should go through an arduous training process ^15^^,^^16^. Therefore, the development of computational strategies using radiological studies to diagnose COVID-19 may help to improve the diagnostic capacity of health systems and support early diagnosis. Additionally, these developments could reduce the high inter-observer variability and rate of false negatives in COVID-19 detection on CT scans.

Some artificial intelligence strategies have been developed to accurately diagnose lung diseases such as pneumonia, pulmonary nodules, chronic obstructive pulmonary disease (COPD), and diffuse pulmonary fibrosis using radiologic studies ^17^^,^^18^. Regarding COVID-19 detection on CT slices, Li, *et al*. ^5^ developed a 3D learning model based on convolutional neural networks (CNN) to perform a differential diagnosis of COVID-19 and other lung diseases on thoracic CT scans ^5^. Silva, *et al*. ^19^ modified the EfficientNetB0 architecture by adding six layers in the feature extraction stage. In contrast, Ragab, *et al.*^20^ proposed a method that combines four CNN and three hand- crafted feature extractors to characterize radiological images exhaustively, features that were then used to train a support vector machine model (SVM) with a cubic kernel. Both methods applied a transfer learning technique using a deep learning model pre-trained on the ImageNet dataset ^21^. Additionally, the authors used standard data augmentation policies, as well as the evaluation scheme proposed by Soares, *et al*. ^22^. However, the authors did not provide enough information to determine if the evaluation stratified by patients the training and testing sets. Avoiding such partitioning might result in over- optimistic results, as pointed out by Silva, *et al*. ^19^. Besides, the low number of cases in different populations and acquisition devices limit the ability of training generalizable supervised deep learning models.

We explored and analyzed here convolutional deep learning representations to support the automatic classification of COVID-19 and non-COVID-19 samples in clinically relevant CT slices previously selected by radiologists. From a supervised scheme, a set of architectures originally trained on the natural image domain were adjusted to implicitly identify radiological visual patterns associated with COVID-19. After, the learned deep representations were used to classify new samples using an end-to-end scheme, as well as high-level embedding vectors with classical machine learning classifiers. Such representations were validated on two different datasets separately showing remarkable results to support radiological analyses. The best performance of the proposed strategies yielded scores of 90% accuracy, 91% sensitivity, and 94% specificity on the mentioned datasets.

## Materials and methods

Thoracic CT is useful to analyze the transverse area, the anatomical structure, and the density of the lungs. Over such images, it is possible to characterize pneumonia from a set of radiological findings as described by the Fleischner Society glossary ^23^^,^^24^. Regarding COVID-19 characterization, there are some predominant findings known to be associated with the disease. The following findings can be considered among the most frequent: bilaterally, lower lobe, peripheral, and basal predominant ground-glass opacities (GGOs) or consolidation with a vascular enlargement ^25^^,^^26^. Besides, GGO is superimposed by a mixed pattern composed of crazy paving, architectural distortion, and perilobular abnormalities ^12^.

The localization of the radiological findings on thoracic CT slices is specific for each patient and varies depending on the stage of the disease ^11^^,^^25^^,^^26^. Hence, a CT-slice selection process was done for better characterization of the COVID-19 patterns. Such selections were manually performed by radiologists exploring the whole CT scans set to determine clinically relevant slices. The datasets used in this work are described in the following section.

### Datasets

Here the evaluation of deep learning representations was considered on two different sets aimed at determining the generalization capability of the classification, as well as the effectiveness in retrospective studies including demographic information on patients. In both cases, only axial CT volumes were considered. The datasets are described as follows:

*SARS-CoV-2 CT Scan dataset.* This public collection contains a total of 210 cases comprising 4,173 thoracic CT slices. A subset of 80 cases corresponds to patients infected by SARS-CoV-2 (2,168 CT slices) and 50 to non-infected patients (757 CT slices). The remaining 80 cases are patients with other pulmonary diseases that were not taken into account in this work. For each CT volume, the most relevant CT slice in terms of radiological findings was manually selected as input for deep learning. This dataset was collected in hospitals of Sao Paulo, Brazil, and all patients were confirmed as positive or negative for SARS-CoV-2 by RT-PCR test (22). Therefore, the automated categorization of patients by the deep learning models may be biased to detect those cases also detected by the RT-PCR test. In our study, this bias was mitigated by the fact that the radiological findings identified by the experts were previously selected on the most significant CT slice for diagnosis.

*FOSCAL dataset.* This dataset comes from a retrospective study of CT scans collected at Clínica FOSCAL in Santander, Colombia, from March 1 to August 19, 2020. The dataset is composed of thoracic CT scans from 355 patients. A subset of 175 patients were positive for COVID-19 infection by RT-PCR (1,171 CT slices) while the remaining 180 patients were negative for COVID-19 (1,364 CT slices) but could have had other pulmonary diseases. Each patient underwent CT and RT-PCR testing for SARS-COV-2. The dataset contains information on 1,846 slices from non-SARS patients and 416 slices from SARS patients. Clinically relevant slices were selected by two radiologists with 3 and 4 years of experience. The number of selected slices per CT scan varies between 4 to 15 from among the tomographies. Every single slice had a spatial size of 512 x 512 pixels. The demographic information and comorbidities distribution are shown in table 1.

**Table 1:** Demographic data and comorbidities distribution of patients included in the FOSCAL dataset

Demographic characteristics	Classes	
	COVID-19	Non-COVID-19
Number of patients	175	180
Number of male/female/unknown	109/66/0	68/96/16
Age [range] (mean ± std)	[6−92] 60.59±18.68)	[6−93] (55.00±17.58)
Comorbidities distribution	46% hypertension	59% no comorbidities
	28% no comorbidities	28% cancer
	15% cardiovascular disease	7% hypertension
	11% cancer	6% others

Our study was retrospective using human subjects’ data and it was approved by the Ethics Committees at *Universidad Industrial de Santander* and of the FOSCAL clinical center in Bucaramanga, Colombia.

**Figure 1:**
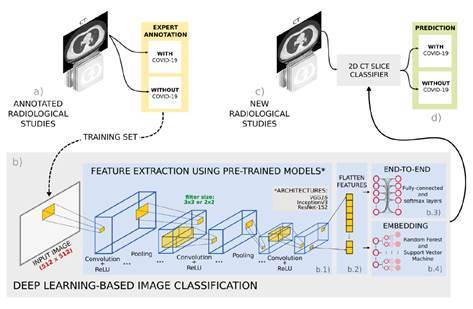
Pipeline of the proposed approach. (**a**) First, a set of radiological studies were collected from different databases with expert annotations. (**b**) Then, a deep learning based strategy was trained to detect COVID-19 cases in three steps: b.1. Different convolutional neural network architectures were tested to characterize the radiological studies; b.2. subsequently, the extracted features were flattened to be used as input for the two proposed classification stages; b.3. an end-to-end approach with fully-connected layer classifier, and (b.4) an embedding approach with machine learning classifiers. (**c**) At the testing stage, new radiological studies were labeled as with or without COVID-19 using the trained models.

We used deep convolutional representations to automatically classify COVID-19 cases from thoracic CT slices. These representations aimed to recover and learn distinctive visual patterns associated with the disease and properly distinguish between COVID-19 and non-COVID-19 CT images. A transfer-learning scheme was implemented to train and adjust the deep representations in an end-to-end classification setup. As an alternative for evaluating deep representations, we took the last fully connected layers as embedding representations, which were then used on classical machine learning classifiers such as random forests or support vector machines. The pipeline of this strategy is shown in figure 1.

### Convolutional neural network architectures (CNNs)

The CT slice characterization by CNNs is based on a hierarchical representation of the visual patterns distinctive of COVID-19 disease and healthy regions. In general, the first layers of the CNN perform a decomposition of the input images into basic visual primitives, which is achieved through a set of kernels learned for the specific task of the convolutional network. Subsequently, more complex patterns are modeled in the upper layers such as relevant texture patterns or regional distributions. For doing so, the activations from the previous decomposition are then convolved with another set of learned filters which extract patterns of a higher degree of non-linear correlation. Finally, such complex patterns are transformed until reaching a semantic level used as a set of features that represent radiological studies with the presence or absence of COVID-19.

Thanks to the success of CNNs on different domains, today there is a wide range of CNN architectures with specific deep properties and learning specifications ^21^. Here we explored three different CNN architectures that are conventional yet representative of the state-of-the-art feature extractors with promising intermediate representations that capture complex visual representations. The architectures herein implemented are:

*VGG16.* The Visual Geometry Group (VGG) developed a relatively deep network composed of 13 convolutional and 3 fully connected layers that account for a total of 138 million parameters. This network is highly uniform around its layers using multiple stacked small-size filters (2 x 2 and 3 x 3) that allow for the learning of more complex features. It was accomplished on the ILSVRC 2014 challenge training and testing with the ImageNet dataset of natural images ^27^.

*ResNet-152.* The Residual Networks consists of a CNN architecture that incorporates identity shortcut connections which reduce the vanishing gradient problem by creating the so-called residual block. Such connections in the image domain improved the classification performance by training deeper networks than the conventional CNN architecture. This network, with 60 million parameters, won the ImageNet Large Scale Visual Recognition Competition (ILSVRC) in 2015 ^28^.

*InceptionV3*. This net is nowadays one of the most representative architectures. Its main proposal centers on reducing the computational cost of deeper networks without affecting generalization through a dimensionality reduction with stacked asymmetric convolutions. First, a 1 x 1 convolution is applied to decrease drastically large filters input dimensions. Then, such large filters are factorized (an N x N filter is the combination of 1 x N and N x 1 filters) and these multiple asymmetric filters are ordered to operate on the same level achieving a progressively wider network instead of a deeper one. With 23 million parameters, InceptionV3 shared first place with the VGG16 on the ILSVRC 2015 challenge ^29^.

### End-to-end classification using transfer learning

The CNN architectures used in this work were selected for their effectiveness in the natural image domain. However, they may be unsuited to represent and differentiate patterns from the radiological domain. Therefore, we obtained an adjusted representation for the radiological domain by using transfer learning. Transfer learning (TL) is a widely known technique that deals with learned weights from large general image representations by adjusting several layers to a specific domain, in our case, CT radiological images. Formally, the learned image representation in the Mk model (k being the ResNet-152, the InceptionV3, or the VGG16) was defined as *M*_*k*_ *= [F,P(F)]*, where *F* is the feature space and *P(F)* the marginal probability distribution. In this case, *F= [F*_*i*_ *(F*_*i-1*_ *)]* represented a hierarchical representation of the general image domain regarding a particular task, *T= [D,M]*. Thus, the task *T*_*t*_ covered the set of classes *D*_*t*_ *= [d*_*1*_ *,...,d*_*n*_ *]* defined in the original problem (ImageNet) ^21^. Then, the aim of TL was to adjust the general codified learning task *T*_*t*_ into a new radiological task *T*_*s*_ as *T*_*t*_ *= [D*_*t*_ *,M*_*t*_ *] → T*_*s*_ *[D*_*s*_ *,M*_*s*_ *]*^30^.

Transfer learning (*T*_*t*_ *→T*_*s*_ ) is an adaptive iterative process learning through several epochs that uses a relatively low learning rate and batches from a new domain, in this case, trained CT slices. Finally, we obtained a deep representation for each CNN architecture with the capacity of capturing COVID-19 patterns on thoracic CT slices.

### Classification from pre-trained deep features

A second option to exploit the pre-trained CNN architectures is to use the embedding vectors to represent input CT images. Then, the feature vectors are used to train classical machine learning models such as random forests and support vector machines. For doing so, the last layer of the CNN nets is flattened into a single vector containing the values associated with different features. The main advantage of this approach is the considerable capacity to characterize complex patterns showing remarkable robustness to distortions, occlusions, and lighting changes ^31^^,^^32^. Additionally, this process reduces the training time and the variability of the results with small datasets ^33^^,^^34^.

Here we applied two classical machine learning algorithms to the computed vectors for the classification task: a support vector machine (SVM) and a random forest (RF).

*Random forest.* An RF defines boundaries in the feature space between the COVID-19 and non-COVID- 19 classes. The RF is comprised of a set of independent decision tree (DT) algorithms. Each DT was trained over different parts of the embedding feature space to reduce prediction variability. A bootstrap aggregating strategy, which consists in randomly selecting a set of training embedding features, was used to build each DT. The final prediction was made by averaging the predictions of the individual trees ^35^. In this process, we obtained B different trees with the ability to predict the disease y.

*Support vector machine:* The SVM selects a hyperplane that separates the embedding features of the two classes. The selection is performed by maximizing the distance between the decision limit and the feature vectors from both classes ^36^. In this work, a polynomial kernel was used to define the decision boundary because this complex classification problem is not linearly separable. The polynomial kernel was defined as: *k(F*_*i*_ *,F*_*j*_ *)=(1+yF* ^*iT*^ *F*_*j*_ *)* ^*d*^ where *F*_*i*_ and *F*_*j*_ are the *i-th* and *j-th* embedded in deep features, *d* is the degree of the polynomial kernel, and gamma is *1/N*^37^.

### Experimental setup

CNNs were previously trained with images from the public ImageNet ^21^ dataset. The resulting weights were used to initialize a new training process with radiological images. The InceptionV3, Resnet-152, and VGG16 models were trained using a batch size of sixteen ^18^, and as an optimization algorithm, we used the Adam algorithm. The learning rate was set to 1e 6 while the loss function was binary cross entropy. The strategies were evaluated in the two mentioned datasets using a cross-validation setup. Each dataset was split into five folds and the experiment was carried out independently. For each fold validation experiment, the respective dataset was partitioned with 80% cases for training and 20% for testing. We also ensured that CT slices of the same patient were in a single fold, i.e., a patient’s CT slices were contained either in the training or in the testing partition. Thus, we guaranteed that the model was dedicated to discriminating among pathologies more than to associating findings from the same patients. In the experiments, we also considered the same number of slices per patient.

The trained models yielded the probability per radiological study of the presence or absence of the disease. A 0.5 probability threshold was used to assign the predicted label 1 (COVID-19) or 0 (non-COVID-19). Here, true positives corresponded to patients effectively classified with COVID-19. It should be noted that the gold standard for COVID-19 diagnosis is the RT- PCR, which has limited sensitivity for its detection. To mitigate the potential bias associated with the sensitivity of the RT-PCR test, for each considered CT volume, CT slice selection was done by an expert radiologist based on observed radiological findings. The evaluation of this classification task was measured by first computing the true positives (TP), true negatives (TN), false positives (FP), and false negatives (FN). The typical metrics for classification tasks such as accuracy (Acc), sensibility (Sens), precision (Pre), and F1 score were used (table 2). The area under the receiver operating characteristic curve (AUC) was also computed.

**Table 2:** Metrics used to evaluate the proposed approach. The metrics are based on the quantification of instances: True positives (TP), false positives (FP), true negatives (TN), and false negatives (FN).

Metric	Formula
Accuracy	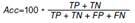
Precision	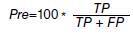
Sensitivity	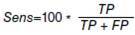
F1 score	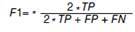

## Results

In this section, we present the performance of the proposed approach separately for each considered dataset and with respect to the two classification schemes.

### Performance for SARS-COV-2 CT scans dataset

Table 3 summarizes the performance for the three methods using the SARS-COV-2 CT scans dataset: a) end-to-end classification from the transfer learning approach using VGG16 and ResNet-152 architectures; b) classification from deep features using SVM and RF methods, and c) a baseline strategy presented by Silva, *et al*. ^19^. The baseline strategy also applied a five-fold cross-validation scheme using the SARS-COV-2 CT scan dataset and separating 80% of the cases (CT scans by patient) for training and 20% for testing in each fold ^19^. Other studies have also used the SARS-CoV-2 CT Scan dataset ^20^^,^^22^, but they are not comparable with ours because they have no precise information regarding the experimentation setup to verify that their training and testing partitions were stratified by cases (patients) as Silva, *et al*. suggest ^19^. The remarkable performance obtained by the VGG16 as regards the accuracy and the AUC metrics should be noted. This could be associated with the small and dense representation kernels on the first layers of this net. The results also suggest that for this data amount, the 16 layers were sufficient to fix a boundary and separate between control and COVID-19 cases.

**Table 3:** SARS-CoV-2 CT Scan dataset average results for the baseline by Silva, et al. (19), end-to-end, and embedding classification approaches. The highest values for each metric across all experiments are highlighted in bold.

Method	Configuration	Acc (%)	Pre (%)	Sens (%)	F1 (%)	AUC (%)
Silva, *et al*. ^19^	EfficientNetB0	86.6 ± 10.1	79.7 ± 20.9	94.8 ± 4.50	-	-
End-to-end	VGG16	92.33 ± 4.81	89.70 ± 6.74	88.96 ± 6.57	89.89 ± 6.38	98.20
	ResNet-152	86.05 ± 1.43	85.52 ± 1.33	76.02 ± 4.01	79.01 ± 3.37	88.51
Embedding	ResNet-152 + RF	90.70 ± 2.80	91.38 ± 2.83	95.62 ± 2.85	93.42 ± 2.38	88.82
	ResNet-152 + SVM	91.40 ± 2.48	95.77 ± 2.83	91.58 ± 2.41	93.63 ± 2.80	91.28

**Figure 2:**
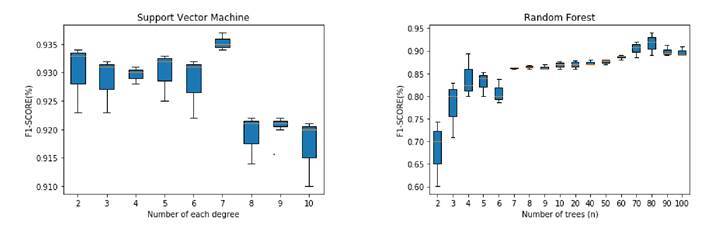
SARS-CoV-2 dataset average results of the embedding with random forest and support vector machine

As for the proposed classification method using the deep features (embedding), we conducted a fine-tuning for the RF and SVM classifiers as shown in figure 2. First, the RF was tuned by varying the number of trees in each iteration taking into account that the maximum depth of the trees was 60. A similar procedure was performed for the support vector machine model using a polynomial kernel that varied the degrees. The best configuration using the RF classifier was for 80 trees with an F1 score of 93.42% while using the SVM classifier it was for 7 degrees of the polynomial kernel achieving an F1 score of 93.63%. These results show that the embedding classification strategy using an SVM classifier obtained better partitions of the deep feature space and the best results in detecting CT slices with COVID-19. All the evaluation metrics for the best configurations of both classifiers are shown in table 3.

Previous results (table 3) showed that the embedding method using an SVM classifier achieved the best performance with an F1 score of 93.63% and precision of 95.77% outperforming the results obtained by Silva, *et al*. ^19^ who only obtained high sensibility. Additionally, the embedding method was quite stable across all the folds: the standard deviation of all performance metrics was less than 2.83% in each metric when comparing it with the transfer learning method and the baseline.

### Performance for the FOSCAL dataset

**Figure 3:**
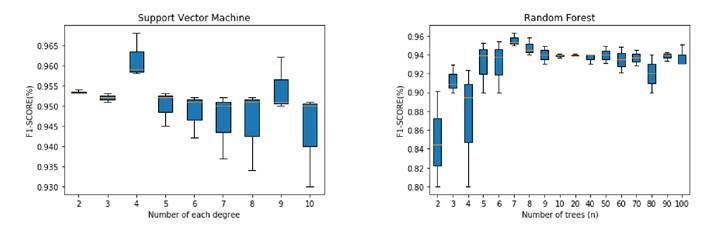
FOSCAL dataset average results of the embedding with random forest and support vector machine

Using the FOSCAL dataset, we evaluated two methods and computed the performance metrics as follows: a) end-to-end classification from transfer learning approach using VGG16, ResNet-152, and InceptionV3 architectures and the classification from deep features using SVM and RF methods. A similar fine-tuning procedure was performed for the embedding method over the FOSCAL dataset shown in Figure 3 by using the same parameters for SVM and RF classifiers. In this case, the best F1 score obtained for the SVM classifier with 6 degrees was 96.46% and for the RF classifier with 7 trees, it was 94.67%.

Table 4 shows the results obtained by the proposed approach using the FOSCAL dataset. With a 95.57% accuracy, a 95.74% precision, a 95.79% sensitivity, and a 95.57% F1 score, the embedding method with the SVM classifier provided a better representation of the embedded space and was able to detect accurately COVID-19 cases on CT slices in the local population. In this case, the VGG16 was also the best net for representing CT slices, which was associated with the amount of data used in the transfer learning scheme. The other deep nets also showed remarkable results on the end-to- end representation achieving general scores up to 90%.

**Table 4:** FOSCAL dataset average results for the end-to-end and embedding classification approache. The highest values for each metric across all experiments are highlighted in bold.

Method	Configuration	Acc (%)	Pre (%)	Sens (%)	F1 (%)	AUC (%)
End-to-end	VGG16	**96.99 ± 1.10**	**96.62 ± 1.21**	**96.61 ± 1.03**	**96.58 ± 1.11**	**99.50**
	ResNet-152	95.57 ± 5.83	95.74 ± 5.53	95.79 ± 5.52	95.57 ± 5.82	98.87
	InceptionV3	94.11 ± 4.45	94.10 ± 4.46	94.08 ± 4.46	94.07 ± 4.50	98.07
Embedding	ResNet-152 + RF	95.11 ± 2.06	94.81 ± 3.56	95.42 ± 2.96	94.67 ± 2.05	96.06
	ResNet-152 + SVM	96.00 ± 2.56	94.74 ± 2.51	96.00 ± 2.12	96.46 ± 1.84	94.15

The evaluation in both datasets showed a remarkable performance of deep representations, which could be key to reducing radiologists’ subjectivity in the analysis and diagnosis of CT scans. Also, the evaluation over both datasets suggested that the best boundaries separation was obtained from embedding vectors with an additional optimization over an SVM hyperparametric space. These embedding vectors recovered a high semantic level of knowledge in deep representations and the additional non-linear kernel separation could induce a better boundary separation among defined diseases.

## Discussion

The main public health problem today in the world is the COVID-19 disease, therefore, it is fundamental to join forces and establish synergies for innovation and propose alternative and complementary methods to characterize, diagnose, and follow up the disease. Such efforts would contribute to early diagnosis, mitigate the collapse of health services, and help with proper analysis and treatment of more patients. Here we presented a deep learning representation for COVID-19 detection in thoracic CT slices. From each CT scan, an expert selected a set of relevant slides exhibiting the most distinctive radiological patterns of COVID-19 patients and healthy lungs. Deep feature extraction was performed to represent the complex visual patterns of the disease exploring different convolution neural networks and then, an end-to-end learning approach and an embedding classification strategy were evaluated to differentiate COVID-19 and non-COVID-19 cases from such deep features. The three networks used (ResNet-152, VGG16, and InceptionV3) achieved an outstanding performance characterizing radiological patterns to detect COVID-19 cases in CT scans. Such deep features were also used to feed two binary classification frameworks: a) end-to-end learning using different CNN architectures, and b) a machine learning approach using SVM and RF models. Finally, these models evaluated new thoracic CT slices to determine whether such image visually corresponded to a lung infected by COVID-19.

The best performance in the open SARS-CoV-2 CT Scan dataset was achieved by the embedding strategy outperforming even state-of-the-art methods evaluated in that dataset. On the other hand, the methods also showed to be capable of identifying positive COVID-19 cases in the FOSCAL dataset. The results also point to their potential implementation in the clinical routine to support the diagnosis. It should be noted that in both datasets, the positive reference was based on the RT-PCR test, which may introduce a bias related to its false positives rate. In both datasets, the CT slice with the most information related to radiological findings was selected to mitigate the potential bias induced by false negatives. Besides, the computational approach was based on a statistical representation that captures visual patterns from a significant amount of data. Hence, it is expected that trained representations would deal with some of the outliers resulting from false negatives annotations.

Currently, several computational strategies have been proposed to detect COVID-19 cases on thoracic CT slices ^19^^,^^20^^,^^22^. Most of these methods have used deep learning based strategies without comparing different network architectures or using public datasets without any additional information about particular conditions of patients, comorbidities, and information on the collection of samples. In contrast, here we used three of the most representative networks to extract deep features. We also evaluated architectures in two different datasets aimed at evidencing the capability of the models to represent COVID-19 patterns from different sources. The performance of these deep features in the binary classification task was evaluated using a typical end-to-end approach and classical machine learning models. Besides, we compared our results and discussed other CNN configurations with the methods used by Silva, *et al*. ^19^ and Ragab, *et al.*^20^ for training and evaluating from the SARS-CoV-2 CT Scan dataset ^22^.

Silva, *et al*. ^19^ proposed a modified EfficientNetB0 architecture. The model was initialized using pre-trained weights from the ImageNet dataset and the newly added layers with normal random values. It was trained with the original images and the resulting transformations of three data augmentation processes, namely rotation, horizontal flip, and scaling. The quantitative evaluation reported by these authors using the SARS-CoV-2 CT Scan dataset showed a 98.99% accuracy, a 99.20% precision, and a 98.80% sensitivity. The validation scheme proposed by other authors ^22^ does not provide enough information to ensure that the training and validation partitions were stratified by patient. Instead, the experimental setup proposed by Silva, *et al*. ^19^ ensured that the training and validation partitions contained different cases (patients). Such setup avoids presenting the CT slices of a particular patient in both partitions and yields a more realistic, yet slightly lower, estimation of the performance ^19^. The authors reported an 86.6% accuracy, a 79.7% precision, and a 94.8% sensitivity. In our approach, an expert selected a set of clinically relevant CT scan slices per patient, and then the partitions set were conformed obtaining a 91.40% accuracy, 95.77% precision, and a 91.58% sensitivity for the embedding classification approach with an SVM classifier. Our results outperform those by Silva, *et al*. ^19^ in two of the performance metrics. In comparison, the deep feature extraction architectures chosen in Silva, *et al.*’s work corresponded to a smaller network (EfficientNetB0 with 5 million parameters) while we used deeper networks (VGG16 and ResNet-152 both have over 60 million parameters). Besides, although the end-to-end learning approach achieved competitive results, the embedding approach exhibited a better boundary to separate the classes thus obtaining the highest performance.

On the other hand, Ragab, *et al.*^20^ used a method combining three handcrafted and four CNN features. These handcrafted features include the discrete wavelet transform (DWT), a gray level co-occurrence matrix (GLCM), and statistical features. As for the CNN architectures, their work fused AlexNet, GoogleNet, ShuffleNet, and ResNet-18 features extractors. The resulting feature vector, with a size of 6948, fed an embedding approach using an SVM classifier to perform the binary classification. Contrarily to Silva, *et al.*’s study and ours, Ragab, *et al.* used the validation scheme they proposed for the SARS-CoV-2 CT Scan dataset ^22^, which seems to allow CT slices of the same patient be both in the validation and training partitions. The results obtained showed very high accuracy, precision, and sensitivity levels, all above 99 %. It is also worth noting that this method used a similar embedding strategy to perform the classification task, but the fused feature vector was more complex and computationally more expensive compared with the single CNN model used in our study. Additionally, we evaluated the RF classifier in the embedding workflow with a correct validation scheme achieving the highest precision, 95.62%, outperforming all the configurations evaluated in this work.

To demonstrate that the method was generalizable to different populations and acquisition devices, we performed an additional evaluation process with data collected locally. The FOSCAL dataset is collected in Santander (Colombia) from different hospitals with diverse CT acquisition devices. The best results were obtained with the end-to-end classification strategy. The strategy yielded a 95.57% accuracy, a 95.74% precision, and a 95.79% sensibility in the FOSCAL testing set. This strategy seems to benefit from the increased number of patients and CT slices available in the FOSCAL dataset. The results show that the method herein proposed was able to accurately detect COVID-19 cases using thoracic CT slices from two different populations and competed well with those from other studies on the state of the art. The proposed strategy is nonetheless dependent on the selection of a significant CT slice, which may limit the automatic detection framework. Moreover, some additional slices with complementary information about radiological findings were discarded and this may be a limitation for including additional information to better discriminate COVID-19 patterns as compared to other classes.

In future studies, we are including the training and evaluation of this method in a cross-dataset setup to ensure the proper COVID-19 disease detection in a larger set of images acquired via CT imaging. In addition, an automatic selection procedure for the clinically relevant CT slices might be a useful tool to facilitate the integration of the proposed strategy in the clinical routine practice. In this sense, the use of an additional stratification related to the stage of the disease could be useful to build and re-train models with more discriminative information. Moreover, the exploration of new deep alternatives may be useful to process the complete CT volumes. In fact, the literature today includes 3D convolutional nets that could be considered in future perspectives to try the problem of automatic COVID-19 diagnosis.
